# Does management reasoning constitute the backbone of the clinical learning environment?: Conceptual analysis of the existing notions

**DOI:** 10.30476/jamp.2020.84431.1138

**Published:** 2021-01

**Authors:** DINESH KUMAR V, ANEESH BASHEER

**Affiliations:** 1 Department of Anatomy, Jawaharlal Institute of Postgraduate Medical Education and Research, Puducherry, India; 2 Department of Medicine, Pondicherry Institute of Medical Sciences, Puducherry, India

**Keywords:** Clinical reasoning, Decision making, Social determinants

## Abstract

Management reasoning is a paradigm whereby learning occurs in a context bound fashion on analysing the biophysical factors existing in the clinical learning environment. In the contemporary medical education forums, much importance is being laid on clinical reasoning and this warrants the appropriate usage of the biomedical knowledge in arriving at the diagnosis. We perceive that clinical reasoning, in pure sense, often doesn’t solve the purpose of rendering the best management plan to the patient. This holds stronger when the case is non-linear and highly complex in nature. Management reasoning fills the gap between hypotheses generation, i.e. accomplishing diagnosis and devising management plan. Indeed, it is a complex activity relying on several factors including the physician’s perceptual abilities and situated cognition derived from formal and informal learning experiences. In contrast to clinical reasoning, which can be taught using structured scenarios, management reasoning necessitates analysing multitude of factors revolving around a patient and prioritizing those in order to titrate the best possible management plan. This commentary spotlights different dimensions of management reasoning, emphasizes the need of teaching it in the current scenarios, enlists the ways it can be taught, and opens the platform for discussing further on this underemphasized topic.

## Introduction

One of the ulterior motives of an academic health care centre is to fortify the clinical learning environment for the students. Designing an optimal clinical learning environment is always an area of discussion among medical educators and accreditors because ineffective training of the students in poor learning environments is detrimental to future patient care ( 1 ). Clinical learning environment can be conceptualized in terms of clinical endeavours performed by the student, the amount he/she learns from them, and factors affecting milieu as such ( 2 ). The Macy Foundation defined the learning environment as, “…social interactions, organizational cultures and structures, physical and virtual spaces that surround and shape participants’ experiences, perceptions, and learning” ( 3 ). Learning from the context and practicing management in real settings are something which could not be learnt by virtue of simulation methods. 

To envisage the complexity in terms of learning environment, let us consider the snapshot of a proceeding in ICU, which is the place of maximum uncertainties and time-sensitive actions in a health care centre ( 4 ). The uncertainties over here arise due to different reasons such as differentiating the plausible diagnoses, choosing the appropriate intervention at the right moment, and prioritizing the available amenities based on the condition of patients. In developing countries, where patients need to shell out of their pockets for meeting the health related expenditures, a physician needs to think of multiple simultaneous management possibilities. It also requires the consideration of psycho-social consequences of therapeutic option alongside keeping the financial factors in mind. Thus, during each clinical encounter, a physician should optimally be able to figure out the precipitants of the current problem and weigh the available management options. 

**Reconsidering the role of physicians in clinical encounters**

Goldszimdt et al. had defined the list of management related tasks which every physician needs to define before planning the action ( 5 ). Initially, he/she should define the exact goal of the intended treatment plan. It might be as simple as treating symptoms in the case of uncomplicated fever, improvement in functioning in a stroke patient, or alteration in prognosis or cure like surgical removal of the inflamed appendix. In certain situations, the need for additional diagnostic tests should be conveyed to the patient because the therapeutic plan might hinge upon it. Next, the psycho-social contexts related to the management plan should be ascertained, and this warrants taking the perspectives of the patient or relatives into account. In addition, the comorbid illnesses of the patient should be considered while deciding upon the management plan, especially when patients need to be put on multiple drugs which tend to interact with each other. The patients should be presented with alternate options, and decision should be made with minimal nudging. Based on the chosen plan, the patient should be adequately educated about the plan and its prognosis. 

Diagnosis and management reasoning can be considered as inter-linked fluidic abilities ( 4 ). Diagnostic reasoning can be made by constellating the history, clinical features and investigation profiles, i.e. either based on pattern recognition or hypothetico-deduction. Management reasoning goes beyond the diagnosis and considers the purview of available infrastructure capacity, cost, insurance coverage, and more. Indeed, while weighing alternative options, the patient’s preferences and shared decision making play an important role rather than the rigid guidelines levied by evidence-based data. Groopman and Hartzband suggested a guided reflection technique for reaching the appropriate diagnosis ( 6 ). By this, a physician should ask himself: What else could this be? Is there something atypical feature that does not fit into the pattern? Could there be more than one diagnosis? In the same line, guided reflection could also be practised for management reasoning by reflecting upon: What is the optimal management strategy for the present case? Can the best treatment be afforded with available infrastructure? Could there be an alternative management strategy which benefits the patient further? 

**Management reasoning and diagnostic reasoning: Close enough but far indeed**

Similar to clinical reasoning, management reasoning can also be considered as a complex process differing between novice and expert. An expert, with considerable years of experience, tend to have patient-centred approach which is duly sharpened by collaborative reasoning and patient empowerment ( 7 ). The collision of perspectives among healthcare professionals also influence the management reasoning, particularly in situations where the therapeutic plans suggested by two different specialists tend to differ. On the other hand, reaching the specific diagnosis has its influence in management reasoning in dual ways. First, upon reaching the specific working diagnosis, the physician could call for specific investigations to confirm it without over-testing; henceforth, the management plan would be precise. Secondly, reaching a vague and non-specific diagnosis would lead to designing of sub-optimal management plan which needs to be rectified subsequently, leading to wastage of resources.

How effective management reasoning could help fortify the patient care? The students learn the diagnostic reasoning and disease management from research evidence, which seldom includes the contextual factors of the patient. While a particular drug which is costlier, but has demonstrated significant outcomes in clinical trials should not be empirically prescribed as such to all subgroups of patients without considering their socio-economic profiles ( 8 ). When a physician misses such contextual factors, it would result in lack of compliance to drug schedule. 

Effective addressing of contextual issues can be considered as the backbone of management reasoning. In practical terms, context can be defined as “the inter-related conditions under which individuals interact with each other and also with the environment”. In other words, the discrete and abstract factors triangulating between the physicians, patient and healthcare environment constitute the context ( 9 ). Weiner et al. ( 10 , 11 ) suggested that a physician should address four sequential questions during every clinical encounter. At first, he should try to find out “contextual red flags”, which could presumably decide whether to proceed with treatment or not. Secondly, he should attempt “contextual probe” of the red flag which should be followed by soliciting the factors which could influence the management protocol and design the individualized care plan. What we need to keep in mind is that there are always multiple pathways for successful management of a specific diagnosis and sometimes even there are multiple acceptable outcomes ( 12 ). 

**Situated cognition in management reasoning**

The pathways required for management reasoning is thus rooted upon situated cognition which is the ability of the physician to perceive and synthesize the particulars of the specific situation ( 13 ). Yet, another theory which could be attributed is ecological psychology, i.e. ability of a physician to interact and filter out the crucial information from the information-rich environment ( 14 ). Applying the principles of situated cognition, we could argue that reasoning abilities are most likely linear in some cases and non-linear in other cases. For example, in diseases such as Colle’s fracture or hydrocele, linearity is likely to occur either because of the straight-forward presentation or lesser degree of variance from typical constructs. However, clinical presentation in an elderly male presenting with anaemia and weight loss is mostly non-linear, warranting the capture of varying patterns in the construct. In such non-linear cases, management is likely to be more challenging because choices have to be made regarding the cost of diagnostic testing, admission of patient, managing the co-morbidities and planning adjunct supportive care. Durning et al. conducted a study to explore the influence of situated cognition in the clinical reasoning abilities of physicians. Initially, they provided the participants with different chief complaints for three common medical diagnoses. Later, they modified the selected contextual factors involving patient, environmental and physician factors and examined the influence of these in the reasoning abilities of the physician. The results of the study showed that contextual factors impacted the expert performance, with small to moderate effects ( 15 ). 

Situated cognition operates in conjunction with cognitive load in terms of processing of the information because the working memory capacity for handling the instructions related to the interventions is always limited ( 16 ). If we consider the fact that a physician needs to process information of varying dimensions, he/she needs to be aware of various elements interacting with each other. It is recommended that the clinical encounter shall be classified depending upon the degree of element interactivity as either high or low. In situations with low element interactivity, the pieces of information can be processed in discretion without consideration of other elements ( 17 ). For example, a patient with lipoma is operated surgically to get rid of swelling. The management of this case seldom warrants taking the other information into consideration. In contrast, when bariatric surgery procedure is be planned in a patient with morbid obesity, his/her wishes or preferences, lifestyle, occupation, and the ability to pay should be considered. This ultimately makes the encounter a high-element interactivity type where all pieces of information, i.e. elements, need to be processed and understood before reaching a management plan ( 18 ). Yet, another concept which operates in the light of management reasoning is ecological psychology, which denotes gathering of critical information regarding the psychology and internal disease states of the patients. In some disease conditions, the information regarding the working/living environment and relationship between family members and community as such might act as crucial pivots for gauging the management plan ( 14 ). 

**Measures for fortifying management reasoning skills in health care profession**

A medical student tends to reason out and make decisions in two domains ( 19 ). In the first domain, thinking is largely non-analytical and a jump into rapid decisions based on learned knowledge or past events. This “intuitive” reasoning might help in linear cases but might culminate as management failures in the case of non-linearity. In contrast, the second domain is reflective and analyzes various elements operating in the context. As mentioned above, using the working memory to assimilate the information is highly demanding in terms of cognitive load ( 20 ). Thus, effective development of management reasoning, which distinguishes the novice from experts, depends upon focused processing of information and shared decision making based on it. To achieve this, we propose initiatives to be developed from two ends: a) student end: keeping in mind the dual processing model and management plan development taking the context into account, and b) monitoring the ongoing interaction between the novice patient and clinical environment. 

The clinical environments differ in terms of their functional milieu and the management reasoning required for achieving the optimal patient care at emergency ward and intensive care units is different from conventional inpatient settings ( 21 ). The linearity of case presentation and addressing of contextual factors also differ. However, the standard flow of action beginning from collecting the critical elements from the history and examination, interpreting the available diagnostic tests, ordering for specific pathognomonic tests, arriving at working diagnosis, and devising an individualized management plan remains the same ( 22 ). 

**Implications and suggestions for incorporating management reasoning**

The development of diagnostic reasoning abilities need not always concur with the development of management knowledge ( 18 ). We could appreciate the difference between students who approach the case solely using his/her biomedical knowledge and experienced physicians who envisage the management as a complete entity ( 23 ). The modern medical education places increased emphasis on clinical reasoning development. Unfortunately, the methodologies and the assessment developed for it are too much “standardized” to maintain authenticity by reducing the noise and variance of the real life contexts. Training for management reasoning should include initiatives for making shared decisions, handling competing priorities of various stakeholders, dealing with uncertainty arising due to contextual constraints, involving unrelated knowledge domains in devising individualized management plans, making patients understand the therapeutic goals, and being fluidic enough to reach acceptable, if not the best, outcomes ( 24 ). Amalgamating the principles of situated cognition and contextual perception along with development of appropriate educational designs to harness non-linearity and complexity science could help the clinical educators to teach management reasoning (Table 1). It is challenging indeed because the measurement is difficult and needs to take into consideration the reasoning process and negotiating the plans over time. However, it is suggested that the clinician educators should understand the salience of management reasoning in the medical education and should bank on it for garnering the utmost benefit of learning from clinical encounters. 

**Table 1:** Suggestions for harnessing management reasoning in clinical learning environment

Restructuring the clinical rounds
Encouraging presentation of cases using the theoretical lenses of ‘management reasoning” by emphasizing on the probabilistic reasoning pathways and uncertainty involved in them.
Acknowledging the contextual constraints and management related uncertainty during audits / rounds.
Discussing the mismanagement episodes / failures incurred and highlighting the cognitive errors behind the mismanagement.
Encouraging critical reflection and developing concept maps for management reasoning.
Incorporating the axioms of medical education such as situated cognition and script development in daily practice.
**Making students recognize the reasoning process as a distinct entity**
Ensuring a non-judgmental environment where trainees are promoted to display their critical thinking and issues handling abilities. This can be done depending on the increasing order of linearity using the principles of complexity science.
Make them consider the alternatives and work on the heuristics / biases possessed by them.
Conducting earmarked sessions on diagnostic / management reasoning and their related errors and if possible, developing it as a competency.
Developing authentic settings, standardized patients and rubrics for measuring the reasoning abilities in such a way that progress of students are periodically monitored.
Making students discuss the difficult cases and failures in seminars / conferences.

## Conclusion

This commentary spotlights different dimensions of management reasoning, emphasizes the need of teaching it in the current scenarios, enlists the ways it can be taught, and opens the platform for discussing further on this underemphasized topic. 
